# Bond-Slip Behavior between Stainless Steel Rebars and Concrete

**DOI:** 10.3390/ma13040979

**Published:** 2020-02-21

**Authors:** Margherita Pauletta, Nicola Rovere, Norbert Randl, Gaetano Russo

**Affiliations:** 1 Polytechnic Department of Engineering and Architecture, University of Udine, Via delle Scienze, 206-33100 Udine, Italy; nicola.rovere@uniud.it (N.R.); gaetano.russo@uniud.it (G.R.); 2 School of Civil Engineering and Architecture, Carinthia University of Applied Sciences, Villacher Str. 1, 9800 Spittal, Austria; n.randl@fh-kaernten.at

**Keywords:** stainless steel, reinforcing bars, bond behavior, tensile concrete, experimental tests

## Abstract

Maintenance of reinforced concrete structures is a prevailing topic, especially with regard to lifeline structures and bridges, many of which are now designed with a service life beyond 100 years. Reinforcement made of ordinary (carbon) steel may corrode in aggressive environments. Stainless steel, being much more resistant to corrosion, is a valid solution to facilitate the protection of the works, increasing the service life and reducing the need for repair and maintenance. Despite the potential for stainless steel to reduce maintenance costs, studies investigating the influence of stainless steel on the behavior of reinforced concrete structures are limited. This study investigated the bond behavior of stainless steel rebars by means of experimental tests on reinforced concrete specimens with different concrete cover thicknesses, concrete strengths, and bar diameters. In each case, identical specimens with carbon steel reinforcement were tested for comparison. The failure modes of the specimens were examined, and a bond stress–slip relationship for stainless steel bars was established. This research shows that the bond behavior of stainless steel rebars is comparable to that of carbon steel bars.

## 1. Introduction

Stainless steel is a viable alternative to carbon for use as reinforcement in reinforced concrete structures. Stainless steel has been proven to be effective in reducing the risk of corrosion, thereby increasing the service life of structures, as well as lowering maintenance and monitoring costs.

Reinforcement made of ordinary steel may corrode when (a) carbonation reduces the alkalinity of the concrete, which can therefore no longer exert its own passivating action and/or (b) the chloride content of the concrete in contact with the reinforcement is higher than a critical threshold. In areas where either of these are likely to occur, the use of ordinary steel reinforcement should be avoided.

Another factor that increases the risk of corrosion of reinforcing steel is extreme heat, as in the case of fire. The high temperature and corresponding internal vapor pressure cause the breakaway of concrete cover, known as spalling [1,2].

The use of stainless steel is particularly advisable for lifelines (critically important structures), such as bridges [3,4], many of which are now designed with a service life beyond 100 years. Indeed, during this long service life design, despite the use of best practices, the phenomena of carbonation may develop or unsuspected chloride levels may be present. Moreover, the good and continuous operation of lifelines is too important to run the risk of having to adopt burdensome or extraordinary maintenance interventions, which, when they concern reinforcement, do not always guarantee a lasting result.

Because stainless steel may cost 6–10 times more than ordinary steel, to contain the cost of the works, it is possible to use both types of steel reinforcements, with stainless steel concentrated in parts of the structure that are most prone to corrosion, such as joints in bridges.

Although the use of stainless steel reinforcement has been recommended in aggressive environments [5,6,7], studies investigating its influence on the bond mechanism between concrete and reinforcement are quite limited [8,9,10,11,12,13].

In [8], the tensile bond strength of stainless steel reinforcements in concrete were compared with conventional steel reinforcement. The comparison of bond test results revealed that the bond strength of the stainless steel bars was comparable to that of conventional ones. 

In [9], the results of an experiment undertaken to evaluate the bond between concrete and flat stainless strips were reported and compared with the behavior of standard carbon steel round reinforcement. The typical pull-out test setup was used. A comparison between the bond behavior of the tested specimens and CEB bond stress–slip relationship [14] was also performed.

In [10], the bond-slip characteristics of corrosion-resistant reinforcing steel bars, including stainless steel bars, embedded in concrete were determined through a beam-end test. The failure modes and the load-slip responses were described. A comparison with carbon steel bars was also provided in this case.

In [11], an experiment was conducted to investigate the influence of bar embedment length and the ratio of duct diameter to bar diameter on monotonic bond-slip response of stainless steel bars embedded in grouted ducts of prefabricated bridge joints. Phenomenological nonlinear bond-slip and end-slip models were developed.

In [12], the bond and anchorage properties of duplex stainless steel bars were studied. A formula for calculating the bond strength of these bars and a formula for anchorage length design were proposed.

In [13], stainless steel application using externally bonded reinforcement (EBR) or near-surface mounted (NSM) bonding techniques was investigated for the structural strengthening of reinforced concrete. The interfacial bond-slip relationships of the NSM and EBR systems were described by means of trapezoidal and power functions. 

In [15,16], experimental investigations were presented to check the feasibility of using stainless steel wire mesh for strengthening concrete circular columns.

The purpose of this research work was to investigate the bond behavior of concrete elements reinforced by means of stainless steel rebars and compare this behavior to that of elements reinforced by conventional carbon steel rebars.

The investigation was carried out by means of experimental tests on specimens with different concrete cover thicknesses, concrete strengths, and bar diameters. The specimens were subjected to tensile stresses parallel to the reinforcement. 

A bond stress–slip relationship for stainless steel bars was established based on the analogous formulation proposed by *fib* Model Code 2010 [17] for carbon steel bars. Such a relationship is useful for analytical solution of the equations governing the bond problem [18,19,20].

The presented results are expected to enrich knowledge about the bond behavior of stainless steel bars embedded in concrete.

## 2. Experimental Investigation

### 2.1. Bond Strength Test Methods: Literature Review

There are various methods to experimentally obtain bond stress–slip relationships. 

**Figure 1:**
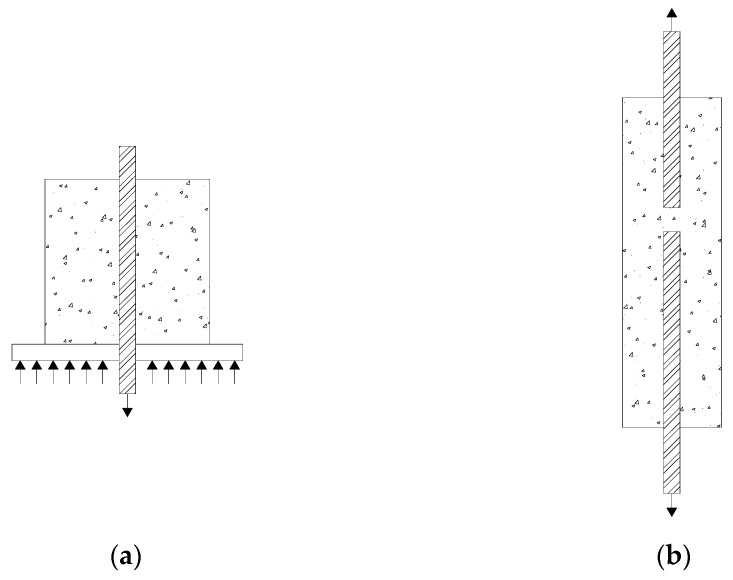
(**a**) Pull-out test with short embedment length; (**b**) modified version of Danish standard pull-out test.

One of the most frequently used method is the pull-out test [21,22]. This is a standard concentric pull-out test (Figure 1a) devised to recreate a uniform distribution of the bond stress along the bar, so a short-bonded length is adopted. Measurements of the pull/push force and the free end displacement of the embedded bar depict uniformly distributed bond stress and local slip. This test setup is very suitable for investigating the effect of various parameters on bond behavior, such as the influence of active or passive confinement (using transverse reinforcement or applying external pressure) [23]. Results obtained from this test have been used to develop a monomial exponential local bond stress–slip relationship [24], similar to the one proposed by *fib* [17]. 

However, many other types of tests have been proposed in the literature in order to meet specific requirements. Among these, a reasonably simple test that permits an accurate measurement of pull-out load and slip displacement was developed [25,26] to simulate the condition where the concrete surrounding the tensile reinforcement is subjected to tensile stresses. This is a modified version of the Danish Standard DS2082 pull-out test [27].

The Danish test requires the concrete surrounding the embedded bar to be confined with spiral reinforcement. This spiral reinforcement is omitted in the modified version (Figure 1b) [27]. The embedded bars are placed end-to-end as in the Danish test in order to eliminate any interaction of forces between them. In some configurations of this test [25,28,29], the embedment length of one bar is longer than that of the other (Figure 1b), and only the slip between the shorter bar and the surrounding concrete is recorded. Meanwhile, in other configurations [26], the two bars have the same anchorage length, and the average of the measured slip between each bar and the surrounding concrete at each end is taken as the corresponding slip of the bar for a particular load.

### 2.2. Test Setup and Instrumentation

**Figure 2:**
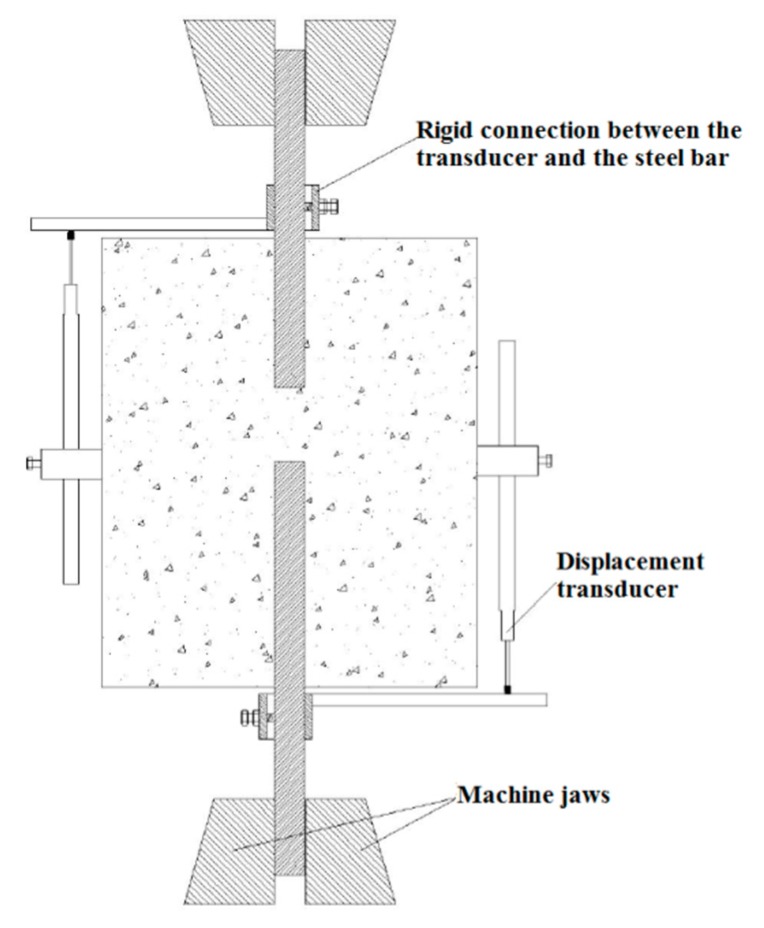
Schematic representation of the test setup.

In this research work, the modified version of Danish standard pull-out test, represented in Figure 1b, was used. A schematic representation of the test setup is shown in Figure 2, where it can be seen that the embedment length of one bar is shorter than that of the other.

For each specimen, two transducers were used to measure the slip of each of the two bars with respect to the concrete during the tensile test. For this purpose, rigid iron supports were mounted on each bar so that the transducer, mounted on the concrete block, could push against the support and measure the relative steel bar–concrete slip. Only the measurements of the short bar were then used to define the bond stress–slip relationship. The bond slip was computed by measuring the total slip and subtracting the elongation of the bar between the transducer and the concrete surface.

Tensile force was applied to the lower bar, while the upper one was kept firm, by a universal MTS 500kN (MTS Systems Corporation, Eden Prairie, MN, USA) machine operated by displacement control. This type of control made it possible to record the softening branch of the force–displacement diagrams. 

Measurements were continued until the force value reached zero.

### 2.3. Specimen Properties

**Figure 3:**
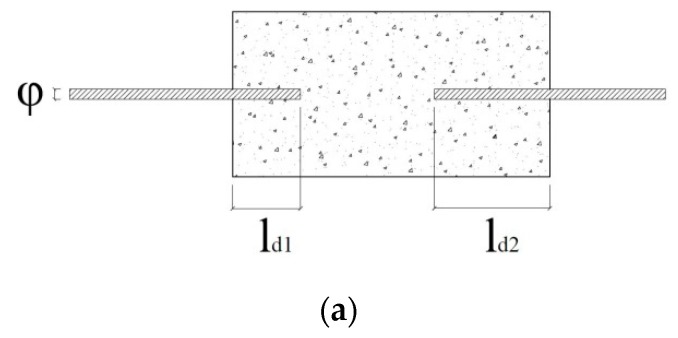

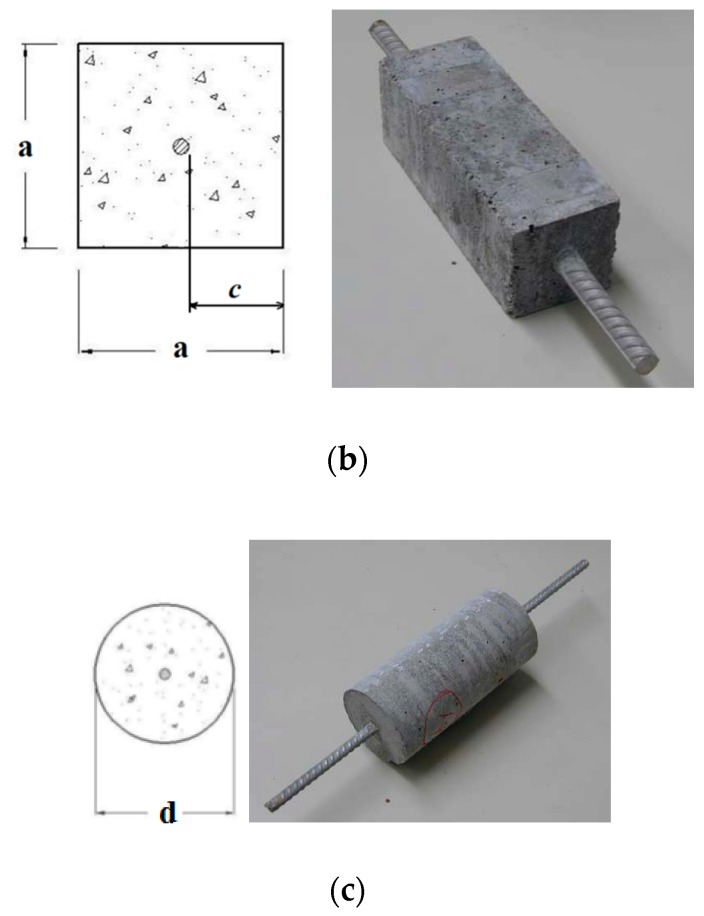
Prismatic concrete specimen: (**a**) longitudinal view; (**b**) square base shape; (**c**) circular base shape.

The specimens consisted of concrete prisms with square or circular base shape (Figure 3), where two reinforcing bars were axially embedded. The embedded ends of the two bars were separated from each other by a distance of 5 cm. The shorter bar length to bar diameter ratio, *l_d_*_1_/*ϕ*, was less than 5 so that it was possible to consider a uniform bond stress distribution along this portion of the bar [17].

The mix design used to produce 1 m^3^ of concrete was as follows:
Aggregates weight 1950 kgCement   340 kgWater   170 L (water/cement ratio = 0.5)


The aggregates were composed of a mix of washed sand (diameter of 0–5 mm) and gravel (diameter of 5–20 mm).

The cement used was a hydraulic binder obtained from the grinding of Portland clinker, natural limestone, and gypsum, with a 28-day strength greater than 32.5 MPa.

The goal of the mix design was to obtain concrete with a cubic characteristic strength equal to 30 MPa. Hence, some samples with different percentages of coarse aggregates, fine aggregates, water, and cement were made and tested in advance to find the best mix design proportions suitable to obtained this strength.

**Figure 4:**
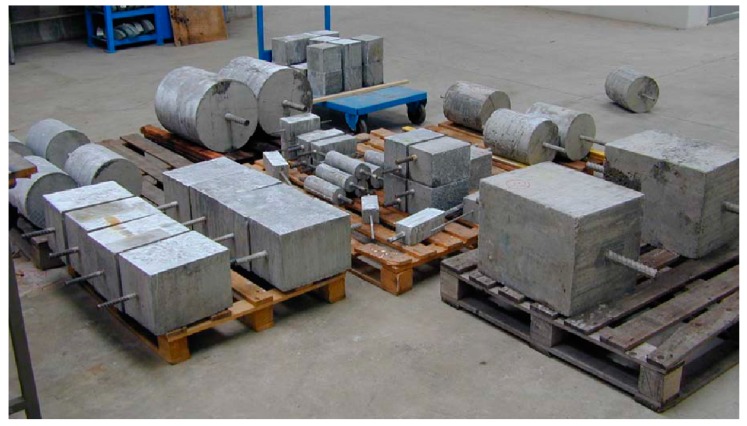
Overview of the realized specimens.

The specimens (Figure 4) were realized in five different castings and numbered S1 to S56. Specimens S15 through to S18 were considered unsuitable for the purpose of this study because the reinforcement axis did not coincide with the concrete axis. Two or more concrete cubic samples were taken from each casting, and they were tested on the same day the corresponding specimens were tested. The mean cubic strength, *R_cm_*, was considered for each group of tested samples, and the mean cylinder concrete compressive and tensile strengths were calculated as *f_cm_* = 0.83·*R_cm_* and *f_ctm_* = 0.3·(*f_cm_*)^2/3^ [17], respectively.

The geometrical characteristics of the specimens are reported in Table 1 from column (2) to (6), where *a* is the square base side length, *d* is the circular base diameter, and *c* is the minimum bar concrete cover. The concrete compressive strength is reported in column (7). For each specimen, the force leading to concrete tensile failure, *F_c,failure_*, was calculated by multiplying the mean concrete tensile strength by the concrete net area (Table 1, column (8)). 

Regarding the rebars, two types of stainless steel—AISI 304 L for 8, 16, and 32 mm diameters and AISI 316 Ti for 16 mm diameter—were used. Only one type of carbon steel, FeB44k, was used. 

**Figure 5:**
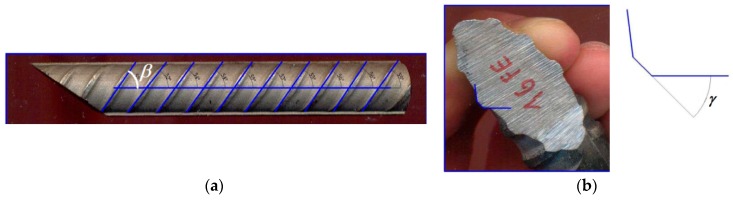
Inclination angles of the ribs: (**a**) angle *β*; (**b**) angle *γ*.

The inclination angle *β* of the ribs with respect to the bar’s longitudinal axis (Figure 5a) and the inclination angle *γ* of the ribs in the bar section perpendicular to the rib direction (Figure 5b) were measured for all the bar diameters. The average of measured values was as follows:

for stainless steel rebars, *β* = 56.9° and *γ* = 41.5°;
for carbon steel rebars, *β* = 52.6° and *γ* = 39°.

To make a comparison between the modified Danish standard test used in this research work, where concrete is subjected to tensile stresses, and the standard pull-out test, where concrete is subjected to compressive stresses, the maximum force values measured for the specimens with carbon steel rebars were compared to the maximum bond force obtained using the maximum bond strength suggested by *fib* [17]. The *fib* strength value is in fact valid for carbon steel rebars subjected to pull-out test. To define this strength, *fib* distinguishes three different confining conditions for concrete: well-confined, when either concrete cover on the bars is larger than 5 times the bar diameter or clear spacing between the bars is more than 10 times the diameter; confined by stirrups; and unconfined. 

The ratios of cover to bar diameter reported in Table 1, column (6), allowed us to identify what specimens could be considered as well-confined and unconfined; no specimen had stirrups.

Moreover, *fib* distinguishes between good or all other bond conditions. *fib* provides that good bond conditions are obtained when

- the bars have an inclination of 45–90° to the horizontal layer during concreting or
- the bars have an inclination less than 45° to the horizontal layer and are up to 250 mm from the bottom or at least 300 mm from the top of the concrete layer during concreting.

Because the specimens described in this study had the bar inclination of 90° during concreting, they were considered to belong to the first case; hence, the bond conditions could be considered as good.

For well-confined concrete and good bond conditions, *fib* suggests the following equation to calculate the maximum bond strength:

(1) $$\tau_{bmax}=k\times \sqrt{f_{cm}}$$

where *k* = 2.5.

For unconfined concrete and symmetrical cover around the bar, the suggested equation for maximum bond strength is the following [17]:

(2) $$\tau_{bu,split}=13.5\times {\left(\frac{f_{cm}}{25}\right)}^{0.25}\times {\left(\frac{l_{d1}}{\phi }\right)}^{-0.45}\times {\left(\frac{25}{\phi }\right)}^{0.2}\times {\left(\frac{c}{\phi }\right)}^{0.25}$$

The force leading to the specimen bond failure, $F_{bmax}$, can be calculated assuming the attainment of the maximum bond strength along the shorter embedment length (*l_d1_* in Figure 3):

(3) $$F_{bmax}=\tau_{max}\times \pi \phi \times l_{d1}$$

where $\tau_{max}$ is equal to $\tau_{bmax}$ (Equation (1)) when the ratio *c*/*ϕ* < 5 and to $\tau_{bu,split}$ otherwise. The forces calculated are reported in Table 1, column (9).

### 2.4. Experimental Results

The maximum force attained by each specimen during the experimental tests, *F_exp_*, is reported in Table 1, column (10).

**Figure 6:**
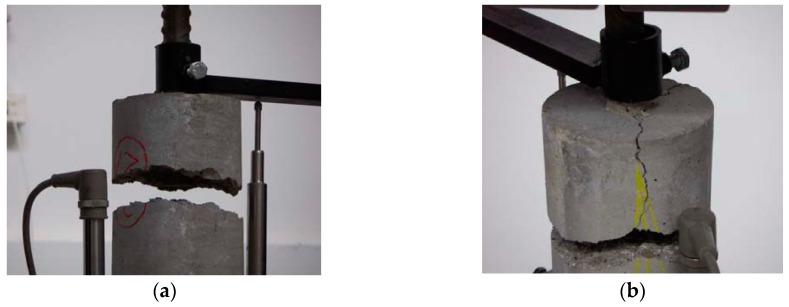
Concrete tensile failure: (**a**) specimen S21; (**b**) specimen S43.

**Figure 7:**
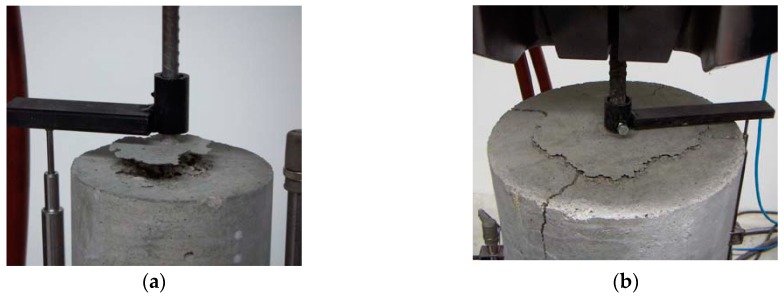
Concrete tensile failure: (**a**) specimen S25; (**b**) specimen S53.

**Figure 8:**
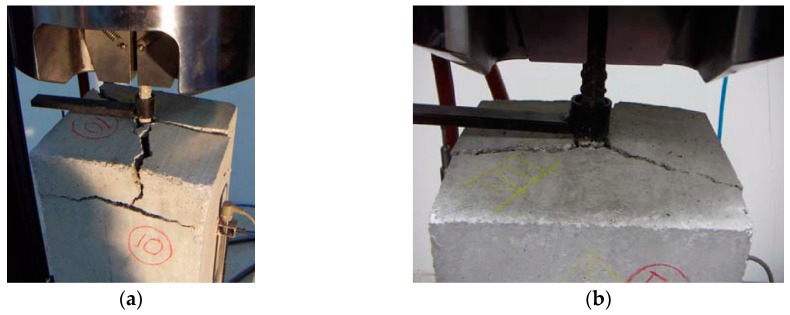
Concrete tensile failure: (**a**) specimen S10; (**b**) specimen S52.

Three failure types were observed:

- concrete tensile failure, which was evidenced by concrete rupture in the specimen section where the longitudinal bars were interrupted, as shown in Figure 6a,b for specimens S21 and S43, respectively; this failure occurred in the specimens with the lowest *a*/*ϕ* or *d*/*ϕ* ratios ;
- pull-out failure, which was evidenced by the shorter bar slipping out of the concrete, accompanied by the dragging of a concrete cone around the bar, as shown in Figure 7a,b for specimens S25 and S53, respectively; this failure occurred only in specimens with the highest *a*/*ϕ* or *d*/*ϕ* ratios; and
- splitting failure, which was evidenced by the radial spreading of longitudinal cracks, which reached the exterior surface of the specimen, as shown in Figure 8a,b for specimens S10 and S52, respectively.

**Figure 9:**
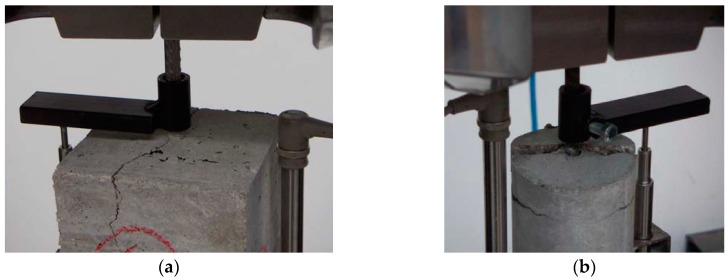
Specimens exhibiting pull-out failure accompanied by splitting failure: (**a**) specimen S7; (**b**) specimen S42.

In some specimens, the slipping of the shorter bar was accompanied by splitting longitudinal cracks. In these cases, the failure was classified as pull-out type if it produced a pull-out cone; otherwise, a splitting failure was assigned. Figure 9a shows specimen S7, to which a pull-out failure type was assigned because the shorter bar slipped out and dragged a concrete cone. Figure 9b shows specimen S42, to which a splitting failure type was assigned because there was no pull-out cone (see Table 1).

The failure types assigned to all the specimens are reported in Table 1, column (11).

**Figure 10:**
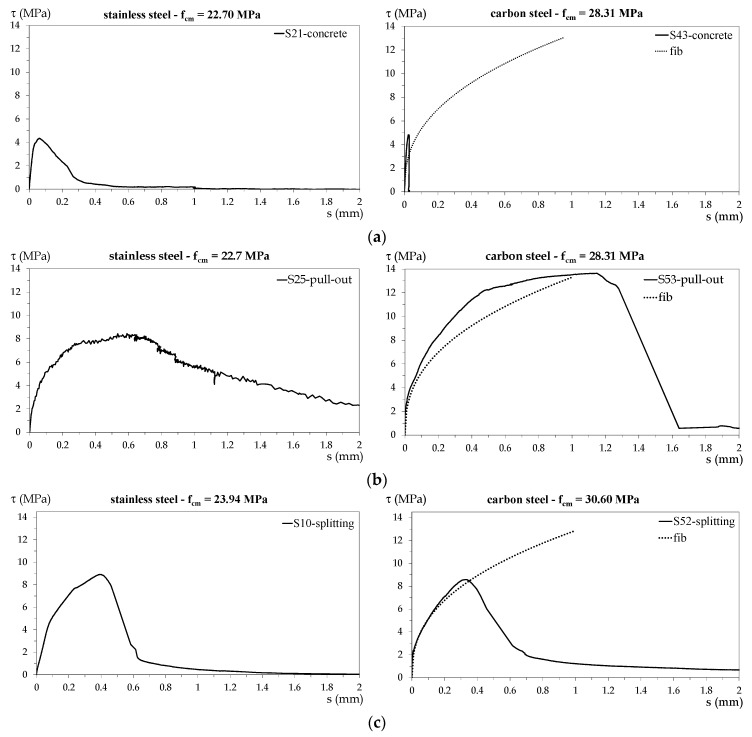

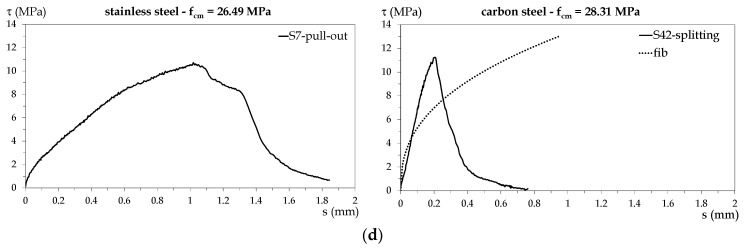
Bond stress–slip diagrams for specimens subjected to different failure types: (**a**) tensile concrete failure for S21 and S43; (**b**) pull-out failure for S25 and S53; (**c**) splitting failure for S10 and S52; (**d**) pull-out failure for S7 and splitting failure for S42.

For each tested specimen, the bond stress–slip (*τ–s*) curve was plotted for the shorter bar considering the bond stress constant along the embedded portion. In Figure 10a–d, the diagrams representing the behavior of the specimens whose failure is shown in Figure 6, Figure 7, Figure 8 and Figure 9, respectively, are reported. 

In the diagrams of specimens with carbon steel rebars, the curves obtained from the *fib* bond stress–slip relationship [17] are plotted for comparison. The *fib* relationship results in a primary increasing branch governed by the following equation:

(4) $$\tau =\tau_{1}\times {\left(\frac{s}{s_{1}}\right)}^{\alpha }$$

where s is the slip, and ($\tau_{1},s_{1}$) are the curve peak coordinates.

In the *fib* relationship, $\tau_{1}$ is given by Equation (1) and

*s*_1_ = 1 mm *α* = 0.4 (5)

The *fib* curves are plotted up to the value of $\tau_{bmax}$ (Equation (1)) for specimens with the ratio *c*/*ϕ* ≥ 5 and up to $\tau_{bu,split}$ (Equation (2)) for specimens with *c*/*ϕ* < 5.

As can be seen in the previous diagrams,

- specimens that exhibited concrete tensile failure (see Figure 10a) attained low slip and bond stress;
- pull-out failure (see Figure 10b) exhibited the most ductile behavior, with high maximum slip and bond stress; and
- curves representing splitting failure (see Figure 10c) showed an ascending branch similar to that caused by pull-out failure up to the peak, while the descending branch indicated a brittle behavior after the peak.

Regarding only the specimens with carbon steel rebars, a comparison was made between the modified Danish standard test using the experimental bond force (*F_exp_* in Table 1, column (10)) and the standard pull-out test using the maximum bond force obtained by *fib* [17] (*F_bmax_* in Table 1, column (9)). The results in Table 1 show that, on average, the bond force developed in the former test was lower than the force developed in the latter. In fact, considering the average of the ratios *F_exp_*/*F_bmax_* (Table 1, column (12)) and excluding the specimens that exhibited concrete tensile failure, the value of 0.69 was obtained. This result confirms that, as expected, the bond strength of a bar anchored in concrete that is subjected to tensile stresses parallel to the bar is less than the bond strength of a bar anchored in concrete that is subjected to compression parallel to the bar.

## 3. Proposed Bond Stress–Slip Relationships

An objective of this investigation was the identification of a relationship based on the *fib* formulation [17] for the primary ascending branch of the bond stress–slip diagrams for the tested specimens. The *fib* relationship is given by the empirical formula in Equation (4), which relates the bond stress τ to the actual slip s to the curve peak coordinates ($\tau_{1},s_{1}$) and to the fixed exponent α, whose values are specified in Equations (1) and (5). It is stressed again that these parameters were obtained from pull-out standard tests [21] for carbon steel rebars in well-confined concrete.

In this work, an attempt was made to adapt the above formula to the case where stainless steel is used in tensile concrete by statistically treating the test results for the specimens with stainless steel rebars. The same analysis was completed for the specimens with carbon steel rebars. To this end, the following steps were taken.

1. The experimental τ=τ(s) curves were smoothed in order to better locate their peak coordinates ($\tau_{1},s_{1}$). For this purpose, each curve was subjected to a simple low-pass filter of the moving average type.
2. For each experimental curve associated with only a pull-out failure, $s_{1}\;\mathrm{and}\;\tau_{1}$ were estimated by locating the first bond stress maximum.
3. The $s_{1}$ values determined in Step 2 were averaged to obtain a common value to be used in subsequent steps.
4. The $\tau_{1}$ values determined in Step 2 were used to evaluate the coefficient k as follows:
  (6) $$k=\frac{\tau_{1}}{\sqrt{f_{cm}}}$$
5. The k values determined in Step 4 were averaged to obtain a common value to be used in subsequent steps.
6. By considering both the curves associated with pull-out failure and the ones associated with concrete and/or splitting failure, a nonlinear regression analysis was performed to estimate the α value from Equation (4) by inserting the values of $s_{1}\;\mathrm{and}\;\tau_{1}$, determined in steps 3 and 4, respectively.

Each analytical step was executed using the software R [30].

The aforementioned analysis was performed separately for the specimens with stainless steel rebars and for the specimens with carbon steel rebars. 

The measured values of parameters $s_{1}$ and *k* are reported in Table 2 for each specimen while values of *α* are not because this parameter was obtained from a nonlinear regression analysis performed on all the acquired points of the experimental curves’ ascending branch. The obtained values of the bond stress relationship parameters for both groups of specimens are also reported in Table 2. For each parameter, the average (AVG), the standard deviation (ST.DEV), and the coefficient of variation (COV = ST.DEV/AVG) of the ratio between the measured and calculated values are reported. 

**Figure 11:**
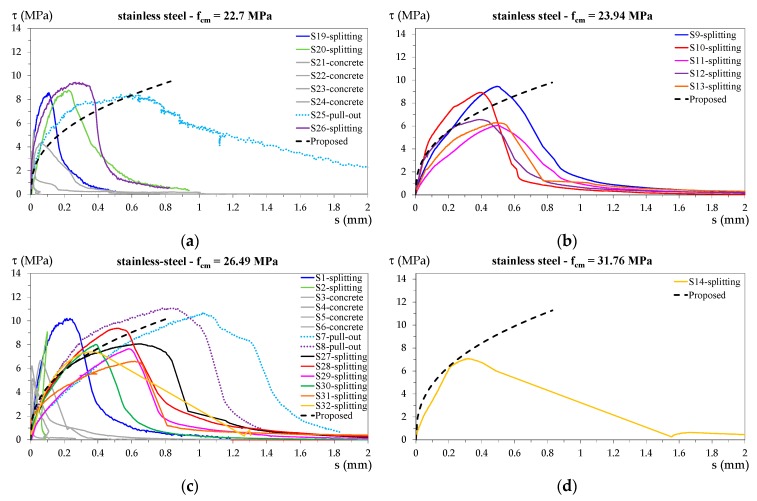
Bond stress–slip diagrams for specimens with stainless steel bars and (**a**) *f_cm_* = 22.70 MPa, (**b**) *f_cm_* = 23.94 MPa, (**c**) *f_cm_* = 26.49 MPa, and (**d**) *f_cm_* = 31.76 MPa.

**Figure 12:**
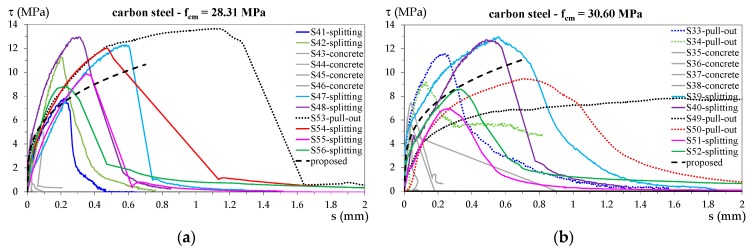
Bond stress–slip diagrams for specimens with carbon steel bars and (**a**) *f_cm_* = 28.31 MPa and (**b**) *f_cm_* = 30.60 MPa.

Figure 11 shows the bond stress–slip diagrams for specimens with stainless steel bars, and Figure 12 shows all the experimental curves for specimens with carbon steel bars. 

The proposed bond stress–slip relationship for each concrete strength is also plotted in the corresponding figures. For both the proposed bond stress relationships, the only varying parameter was $\tau_{1}$, which depended on $f_{ck}$; therefore, in these figures, the curves relating to specimens with the same concrete cylinder strength are plotted in separate diagrams. In this way, a direct comparison with the proposed relationships is possible. Moreover, to make a comparison between individual specimens, the following curve colors have been used:

- blue for specimens S1, S19, S33, and S41;
- light green for specimens S2, S20, S34, and S42;
- light blue for specimens S7, S25, S39, and S47;
- purple for specimens S8, S26, S40, and S48;
- black for specimens S9, S27, S49, and S53;
- red for specimens S10, S28, S50, and S54;
- fuchsia for specimens S11, S29, S51, and S55;
- green for specimens S12, S30, S52, and S56;
- orange for specimens S13 and S31;
- dark yellow for specimens S14 and S32.

The curve line for specimens that exhibited pull-out failure is dotted, while that for specimens that exhibited splitting failure is continuous.

Specimens that exhibited concrete tensile failure are all represented by grey continuous lines.

## 4. Discussion and Modification

**Figure 13:**
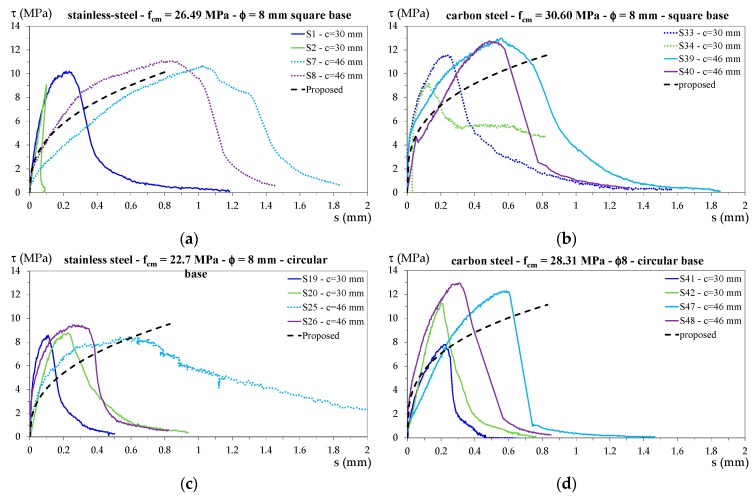
Bond stress–slip diagrams for specimens with different cover: (**a**,**c**) specimens with stainless steel bars; (**b**,**d**) specimens with carbon steel bars.

With reference to the values in Table 2 and Figure 11 and Figure 13, the following observations can be made.

1. For both groups of specimens, the value proposed for parameter *k* resulted in an accurate prediction of the bond strength because the AVG of the ratio of measured to proposed values was practically equal to 1 for both specimen types; moreover, the predictions were precise with COV of 0.106 for specimens with stainless steel rebars and COV = 0.207 for specimens with carbon steel bars. The *k* value, equal to 2 for both specimen types, was 20% lower than the one proposed in the *fib* relationship (Equation (1)), which is valid for concrete under compressive stress.
2. A wider range of values was observed for parameter *α*, which represents the slope of the bond stress–slip curve because the COV value exceeded 0.3 for both types of specimens. Regarding this parameter, it should be noted that, on average, the specimens with thinner cover *c* exhibited bond stress–slip curves with higher stiffness (steep curve slope), while specimens with greater cover exhibited curves with a lower stiffness (lower slope). This can be appreciated by comparing specimens that were identical in all characteristics apart from the cover, such as those reported in Figure 13. However, a differentiation of parameter *α* on the basis of the cover seems to be an unnecessary complication considering that not even the *fib* relationship accounts for it. For parameter *α*, the relationship described herein proposes the same value as that proposed in the *fib* relationship (*α* = 0.4) for specimens with stainless steel rebars (*α* = 0.4) and a lower value (*α* = 0.3) for specimens with carbon steel rebars.
3. Regarding parameter $s_{1}$, the COV of the ratio of measured to proposed values for specimens with stainless steel rebars was fairly low (COV = 0.240), while it was high (COV = 0.829) for specimens with carbon steel rebars. Considering the specimens with the highest COV and their test results reported in Table 2 and Figure 12b, it is apparent that specimen S49 exhibited singular behavior, different from that of all the others, which resulted in a very high $s_{1}$ value. Conversely, specimen S34 resulted in a very low $s_{1}$ value among specimens exhibiting pull-out failure. Moreover, as already observed for parameter *α*, the carbon steel specimens with thinner cover *c* exhibited bond stress–slip curves different from those of specimens with greater cover. In particular, the measured slip $s_{1}$ was lower in specimens with thinner cover, as can be seen in Figure 13b. The phenomenon underlines the observation that greater cover results in more ductile behavior. By excluding specimens S49 and S34 and removing their $s_{1}$ values from the average, the obtained average $s_{1}$ value was 0.64; *k* remained approximately equal to 2, and the new value of *α* was around 0.4.
4. On the basis of the previous evaluation, excluding the outliers for specimens with carbon steel rebars, the result was *α* being equal to the value proposed by the *fib* relationship (*α* = 0.4) for both types of specimens. However, the $s_{1}$ values obtained in this work were lower than the value indicated for the *fib* relationship (Equation (5)). The finding on $s_{1}$ was to be expected as the tested specimens had concrete subjected to tensile stresses, which resulted in less ductile behavior compared to concrete subjected to compressive stresses, as for the *fib* relationship.
5. On the whole, it can be said that the bond behavior of the stainless steel rebars was quite similar to that of the carbon steel ones. The differences, mainly regarding the peak slip value $s_{1}$, might have been due to the different angle of inclination of the ribs and/or the different type of material. However, given the scattering of the test results, it is difficult to determine which of these two parameters had the greater influence.

By considering the above observations, the parameters of the bond stress–slip relationships that may be used for stainless steel and carbon steel rebars embedded in tensile concrete are reported in Table 3.

## 5. Conclusions

The bond behavior of both stainless steel and carbon steel rebars embedded in concrete subjected to tensile stresses were analyzed by performing a series of 52 tests on specimens and considering the variables of concrete cover, concrete strength, and bar diameter. The authors make the following conclusions.

1. A comparison between the modified Danish standard test (tensile concrete) and the standard pull-out test (compression concrete) for carbon steel rebars highlights the fact that the bond strength of a bar anchored in concrete subjected to tensile stresses parallel to the bar is less than the bond strength of a bar anchored in concrete subjected to compression parallel to the bar. In this research, the average of the ratios *F_exp_*/*F_bmax_* (Table 1, column (12)) was equal to 0.69.
2. The analogous comparison in terms of peak slip, $s_{1}$, corresponding to the peak bond stress, $s_{1}$, shows that the behavior of carbon steel rebars embedded in tensile concrete is less ductile than that of rebars embedded in compression concrete, with the $s_{1}$ value for concrete in tension (0.64 mm) being lower than the value for concrete in compression (1 mm).
3. The comparison between stainless steel bars and the carbon steel ones shows that the bond stress–slip parameters for the two types of reinforcements are similar; hence, the difference in the material does not significantly affect the bond behavior.
4. For both stainless steel and carbon steel rebars, both the slip corresponding to the peak bond stress and the slope of the bond stress–slip relationship show a dependence on the concrete cover, with the peak slip being lower for the thinnest covers and the slope of the stress–slip curve being steeper. On the whole, a greater cover produces a more ductile behavior.

The present study enriches the database of bond tests performed on stainless steel rebars available in the literature and proposes a new bond stress–slip relationship for this type of reinforcement.

## Figures and Tables

**Table 1:** Characteristics of the specimens and experimental results.

(1)	(2)	(3)	(4)	(5)	(6)	(7)	(8)	(9)	(10)	(11)	(12)
Specimen -	*a* or *d*	*c*	*l_d1_*	*ϕ*	*c/ϕ*	*f_cm_*	*F_c,failure_*	*F_bmax_*	*F_exp_*	Failure	*F_exp_/F_bmax_*
	(mm)	(mm)	(mm)	(mm)	-	(MPa)	(N)	(N)	(N)	type	-
SPECIMENS WITH CARBON STEEL REBARS											
square base shape											
S33	68	30	24	8	3.8	30.6	10.51	15.14	7.00	pull-out	0.46
S34	68	30	26	8	3.8	30.6	10.51	14.60	5.59	pull-out	0.38
S35	76	30	48	16	1.9	30.6	12.72	11.08	14.94	concrete	
S36	76	30	48	16	1.9	30.6	12.72	11.08	17.89	concrete	
S37	80	30	60	20	1.5	30.6	13.83	10.02	18.47	concrete	
S38	80	30	60	20	1.5	30.6	13.83	10.02	21.66	concrete	
S39	100	46	40	8	5.8	30.6	22.90	13.90	13.06	splitting	0.94
S40	100	46	40	8	5.8	30.6	22.90	13.90	12.84	splitting	0.92
S49	220	102	80	16	6.4	30.6	110.98	55.61	31.55	pull-out	0.57
S50	220	102	80	16	6.4	30.6	110.98	55.61	38.04	pull-out	0.68
S51	220	100	100	20	5.0	30.6	110.65	86.89	43.56	splitting	0.50
S52	220	100	100	20	5.0	30.6	110.65	86.89	53.92	splitting	0.62
circular base shape											
S41	68	30	24	8	3.8	28.31	9.98	14.85	4.71	splitting	0.32
S42	68	30	24	8	3.8	28.31	9.98	14.85	6.79	splitting	0.46
S43	76	30	48	16	1.9	28.31	12.08	10.87	11.66	concrete	
S44	76	30	48	16	1.9	28.31	12.08	10.87	12.69	concrete	
S45	80	30	60	20	1.5	28.31	13.13	9.83	18.22	concrete	
S46	80	30	60	20	1.5	28.31	13.13	9.83	16.81	concrete	
S47	100	46	40	8	5.8	28.31	21.75	13.37	12.35	splitting	0.92
S48	100	46	40	8	5.8	28.31	21.75	13.37	13.01	splitting	0.97
S53	250	117	80	16	7.3	28.31	136.23	53.49	54.84	pull-out	1.03
S54	250	117	80	16	7.3	28.31	136.23	53.49	48.37	pull-out	0.90
S55	250	115	100	20	5.8	28.31	135.91	83.58	62.02	splitting	0.74
S56	250	115	100	20	5.8	28.31	135.91	83.58	55.34	splitting	0.66
									AVG		0.69
SPECIMENS WITH STAINLESS STEEL REBARS											
square base shape											
S1	68	30	24	8	3.8	26.49	12.2		6.16	splitting	
S2	68	30	26	8	3.8	26.49	12.2		5.94	splitting	
S3	76	30	48	16	1.9	26.49	14.9		16.20	concrete	
S4	76	30	48	16	1.9	26.49	14.9		15.00	concrete	
S5	80	30	60	20	1.5	26.49	16.2		17.84	concrete	
S6	80	30	60	20	1.5	26.49	16.2		19.32	concrete	
S7	100	46	40	8	5.8	26.49	26.5		10.78	pull-out	
S8	100	46	40	8	5.8	26.49	26.5		11.15	pull-out	
S9	220	102	80	16	6.4	23.94	120.1		38.01	splitting	
S10	220	102	80	16	6.4	23.94	120.1		35.88	splitting	
S11	220	100	100	20	5.0	23.94	119.8		53.77	splitting	
S12	220	100	100	20	5.0	23.94	119.8		41.24	splitting	
S13	350	159	160	32	5.0	23.94	303.3		101.12	splitting	
S14	350	159	160	32	5.0	31.76	366.1		113.92	splitting	
circular base shape											
S19	68	30	24	8	3.8	22.70	11.0		5.17	splitting	
S20	68	30	24	8	3.8	22.70	11.0		5.40	splitting	
S21	76	30	48	16	1.9	22.70	13.4		10.48	concrete	
S22	76	30	48	16	1.9	22.70	13.4		13.13	concrete	
S23	80	30	60	20	1.5	22.70	14.6		13.59	concrete	
S24	80	30	60	20	1.5	22.70	14.6		11.76	concrete	
S25	100	46	15	8	5.8	22.70	23.9		3.18	pull-out	
S26	100	46	25	8	5.8	22.70	23.9		5.93	splitting	
S27	250	117	80	16	7.3	26.49	166.1		32.47	splitting	
S28	250	117	80	16	7.3	26.49	166.1		37.70	splitting	
S29	250	115	100	20	5.8	26.49	165.8		48.13	splitting	
S30	250	115	65	20	5.8	26.49	165.8		32.75	splitting	
S31	400	184	160	32	5.8	26.49	424.4		106.14	splitting	
S32	400	184	160	32	5.8	26.49	424.4		119.01	splitting	

**Table 2:** Parameters of the bond stress–slip relationships obtained from statistical processing.

Specimen	*s*_1_	*k*	*α*
-	(mm)	-	-
Stainless steel			
	measured values		
S7	1.027	2.060	
S8	0.832	2.147	
S25	0.630	1.746	
Calculated values	0.83	2	0.4
AVG (measured/proposed)	1.000	0.992	1.055
ST.DEV (measured/proposed)	0.240	0.106	0.393
COV (measured/proposed)	0.240	0.106	0.393
Carbon steel			
	measured values		
S33	0.229	2.081	
S34	0.125	1.633	
S49	1.641	1.416	
S50	0.704	1.709	
S53	1.142	2.562	
S54	0.469	2.254	
Proposed values	0.7	2	0.3
AVG (measured/proposed)	0.975	1.029	0.952
ST.DEV (measured/proposed)	0.808	0.213	0.327
COV (measured/proposed)	0.829	0.207	0.343

**Table 3:** Parameters of the proposed bond stress–slip relationships.

Steel Type	*s*_1_ (mm)	*k*	*α*
Stainless steel	0.83	2	0.4
Carbon steel	0.64	2	0.4
